# Work, travel, or leisure: comparing domain-specific physical activity patterns based on rural–urban location in Canada

**DOI:** 10.1186/s12889-023-16876-1

**Published:** 2023-11-10

**Authors:** Chelsea Pelletier, Nicole White, Annie Duchesne, Larine Sluggett

**Affiliations:** 1 School of Health Sciences, Faculty of Human and Health Sciences, University of Northern British Columbia, 3333 University Way, Prince George, British Columbia V2N 4Z9 Canada; 2 Department of Family Practice, Faculty of Medicine, University of British Columbia, Vancouver, Canada; 3 Department of Psychology, Faculty of Human and Health Sciences, University of Northern British Columbia, Prince George, Canada; 4 University of Northern British Columbia, Prince George, Canada

**Keywords:** Physical activity behavior, Correlates, Exercise, Rural health, Social determinants of health, Sex differences, Active transportation, Occupational physical activity, Recreation, Leisure-time physical activity, Physical activity domains

## Abstract

**Background:**

Physical activity occurs across various domains including leisure/recreation, for transportation, or for work or household reasons. Rural and urban active living environments are characterized by different opportunities for physical activity within each domain which may translate into different patterns of behavior. The aim of this study was to compare rural–urban differences in physical activity across different domains, and explore interactions between sociodemographic factors, physical activity domains, and rurality.

**Methods:**

We used self-reported data collected across three physical activity domains (active transportation, recreation, occupational/household) and relevant sociodemographic variables from the Canadian Community Health Survey. Adjusting for sociodemographic factors, we did two separate cross-sectional analyses: 1) binary logistic regression to determine the odds of reporting any activity in each domain, and 2) ordinary least squares regression using the sub-samples reporting > 0 min per week of activity to compare how much activity was reported in each domain.

**Results:**

Our final survey weighted sample of Canadian adults (mean age 47.4 years) was *n* = 25,669,018 (unweighted *n* = 47,266). Rural residents were less likely to report any active transportation (OR = 0.59, 95% CI [0.51, 0.67], *p* < .0001). For recreational physical activity, rural males had lower odds (OR = 0.75, 95% CI [0.67, 0.83], *p* < .0001) and rural females had higher odds (OR = 1.19, 95% CI [1.08, 1.30], *p* = .0002) of reporting any participation compared to urban residents. Rural males (OR = 1.90, 95% CI [1.74, 2.07], *p* < .0001) and females (OR = 1.33, 95% CI [1.21, 1.46], *p* < .0001) had higher odds of reporting any occupational or household physical activity.

**Conclusions:**

Urban residents tend to participate in more active transportation, while rural residents participate in more occupational or household physical activity. Location-based differences in physical activity are best understood by examining multiple domains and must include appropriate sociodemographic interactions, such as income and sex/gender.

**Supplementary Information:**

The online version contains supplementary material available at 10.1186/s12889-023-16876-1.

## Introduction

Rural populations have higher rates of preventable mortality [1–3] and are less likely to meet physical activity guidelines than people living in urban settings [4]. The rural–urban discrepancy in physical activity behavior is attributed to reduced access to dedicated facilities and spaces [5], neighbourhood walkability and active transportation barriers [6], and lack of access to supports enabling participation (e.g., childcare, [7]). As place is an important element of an intersectional approach to understanding behavior [8], adopting a place-based analysis of physical activity behavioral patterns is necessary to understand how people are active in their everyday lives and to support an equity approach to physical activity promotion.

Physical activity occurs in a variety of settings for many different purposes. Broad physical activity domains include occupational, household, travel/transportation, or leisure time (e.g., sport, recreation). Each of these domains of activity contribute to health in different ways [9] and are uniquely influenced by sociodemographic and environmental factors [10, 11]. For instance, in low-income countries, people meet physical activity guidelines by engaging in relatively more work/household activity, and in high-income countries, people report relatively more leisure-time physical activity [12]. Studies comparing physical activity across rural and urban populations tend to focus on a single component of physical activity (e.g., leisure time; [13]) or consider a composite model across all domains based on meeting physical activity guidelines (e.g., [4, 14]). It is important to consider domain-specific patterns in physical activity across the rural–urban continuum as there may be differences in the ways urban and rural residents meet physical activity guidelines across domains. Recent studies in the United States have found rural–urban differences in physical activity are eliminated when considering occupational or household activities as rural residents spend more time engaged in household physical activity with less time in active transportation [15–17]. Differing patterns in physical activity are associated with an inverse relationship between urbanization level and occupational physical activity while opportunities for active transportation are closely related to population density, infrastructure, and social norms [12, 18]. By focusing on leisure time activity or a composite measure across all domains of activity, data may be misrepresenting or not properly capturing physical activity behavior in rural communities. Understanding how and why people engage in physical activity across different domains is required to locate where specific differences occur and develop targeted intervention strategies.

Our previous research demonstrated a location by sex interaction best predicted rural–urban variation in physical activity in Canadian adults, wherein rural females were less active than urban females, and rural males were more active than urban males [4]. As rural and urban populations tend to differ on several sociodemographic correlates of physical activity (e.g., income, educational attainment), analyses of physical activity behavior must consider an intersectional lens, incorporating multiple confounding influences across sociodemographic factors and place. Focusing solely on rural–urban location-based differences may miss the nuance and contextual factors associated with physical activity behavioral patterns. Given the relatively small population size of rural communities and differences in the context and definitions of rurality between jurisdictions, country-level nationally representative samples are required to enable multi-level analyses considering interactions between domain-specific physical activity behavior, sociodemographic correlates, and place-based variables.

The aim of this study was to investigate rural–urban differences in the distribution of physical activity behavior across different domains of activity in a nationally representative sample of adults living in Canada. We further aimed to explore interactive effects of rural–urban location and sociodemographic correlates on domain-specific physical activity.

We predicted rural residents would report lower odds of participation and fewer minutes per week of active transportation and recreational physical activity compared to urban residents. We predicted rural residents would have higher odds of reporting any minutes per week and more minutes per week of occupational/household physical activity compared to urban residents.

## Methods

### Data source

We used data from the 2017 cycle of the Canadian Community Health Survey (CCHS), an annual cross-sectional survey providing a representative sample of the health status and health determinants of the Canadian population. The CCHS covers approximately 98% of the Canadian population over 12 years of age, excluding individuals living on Indigenous reserves and Crown Lands, full time members of the Canadian Forces, institutional residents, youth in foster care, and residents of other remote regions. For the 2017 cycle, approximately 58,600 computer-assisted interviews were conducted over telephone or in person, representing a response rate of 62.8%. Each person who participated in the survey was assigned a sampling weight used to provide a nationally representative sample [19].

We excluded youth participants (age < 18, *n* = 4410), individuals who were currently pregnant or who did not answer (*n* = 588), and individuals with missing data (refusal or not stated responses) or an answer of “don’t know” on any variable of interest (*n* = 4686; see Supplementary file for comparison of included and excluded participants). Our final survey-weighted sample was *n* = 25,669,018 (unweighted *n* = 47,266). Data was accessed at the University of Northern British Columbia Research Data Centre following approval as regulated by Statistics Canada.

### Variables

#### Physical activity domains

We selected three variables to index domain-specific physical activity (minutes per week): time spent engaged in active transportation, recreational activity, and occupational/household-related physical activity (Table 1).

**Table 1:** Variables used in analysis [19]

CCHS Variable	Definition	Intensity
PAADVTRV – active transportation	In the last 7 days, that is from last [day of the week 7 days ago] to yesterday, did you use active ways like walking or cycling to get to places such as work, school, the bus stop, the shopping centre or to visit friends?” How much time in total, in the last 7 days, did you spend doing these activities? Please only include activities that lasted a minimum of 10 continuous minutes	N/A
PAAVREC – recreation	[Not including activities, you just reported,] [i.e. on active transport] in the last 7 days, did you do sports, fitness or recreational physical activities, organized or non-organized, that lasted a minimum of 10 continuous minutes? Examples are walking, home or gym exercise, swimming, cycling, running, skiing, dancing and all team sports	Did any of these recreational physical activities make you sweat at least a little and breathe harder? In the last 7 days, how much time in total did you spend doing these activities that made you sweat at least a little and breathe harder?
PAADVOTH – occupational/household	In the last 7 days, did you do any other physical activities while at work, in or around your home or while volunteering? Examples are carrying heavy loads, shoveling, and household chores such as vacuuming or washing windows. Please remember to only include activities that lasted a minimum of 10 continuous minutes	Did any of these other physical activities make you sweat at least a little and breathe harder? In the last 7 days, how much time in total did you spend doing these activities that made you sweat at least a little and breathe harder?

#### Rural–urban location

The CCHS includes several geographical variables recording participants’ area of residence derived from postal codes and census-based geographical boundaries (e.g., population density). We used a dichotomous (binary) indicator of rurality categorizing location as urban or rural for this study. A population centre (urban) was defined as having a minimum population concentration of 1000 with a population density of at least 400 people per square kilometre, while a rural area was defined by a population of fewer than 1000 people or a population density below 400 people per square kilometre. The CCHS provides a four-level variable (rural area, small population centre, medium population centre, large urban centre) and a seven-level variable corresponding to census metropolitan influence zones. The binary categorization of rurality was chosen for this study after preliminary analyses indicated all three variables captured similar patterns of physical activity behavior across rurality designations and little information was added by increasing the resolution of community size and density.

#### Sociodemographic characteristics

Sociodemographic characteristics were collected as part of the standard CCHS questions and treated following procedures we have previously reported [4]. Participants self-identified their sex as male or female (coded male = 0, female = 1). Age was self-reported and treated as a grand-mean-centred continuous measure. The selected BMI variable was derived from self-reported height and weight, corrected for self-report, and grand-mean-centred for analysis. A three-level variable was used to describe participants’ education: less than secondary school graduation/secondary school graduation/post-secondary certificate, diploma, or university degree (coded as secondary school graduation [mode] = 0). Self-reported household income was collapsed into roughly equal weighted quintiles: $0–29,999; $30,000–59,999; $60,000–99,999; $100,000–149,999; ≥ $150,000 (coded as $60,000–99,999 = 0). Perceived health status was reported across five levels, ranging from poor to excellent (coded as Good = 0). Sense of belonging was reported across four levels from Very Strong to Very Weak (coded as Somewhat Strong [mode] = 0). Sense of belonging was included as a variable of interest based on previous work establishing the relationship between community connectedness and physical activity for rural communities [20].

The CCHS sampling strategy is equally subdivided into four data collection periods: 1) January to March (used to anchor regression analysis); 2) April to June; 3) July to September; and 4) October to December.

### Data analysis

Data analysis was conducted using R 3.4.3 and packages arsenal [21] and survey [22]. Survey weights provided by Statistics Canada were employed to ensure outcomes would be representative of the Canadian population and bootstrap replicate resampling weights were employed for variance calculations.

In all analyses, a model-fitting approach was employed to determine which factors best explained variance in physical activity participation. Location was always retained in the best-fitting model. Other terms were retained in final models if they significantly improved overall model fit. Model fit was compared with log-likelihood ratio tests, using a cut-off of *p* < 0.05 to determine whether specific variables significantly contributed to improving the fit of the overall model.

For each outcome, we computed a base model to assess the bivariate relationship between rural–urban location and activity. Next, we fitted a fully adjusted model with all identified sociodemographic factors as covariates, which were then sequentially removed in order of smallest t-value to determine the best-fitting covariate model, defined as the model for which no further terms could be removed without significantly reducing the goodness of fit of the model. Surviving covariate terms were then estimated in 2-way interactions with location to assess whether the relationship between rural–urban location and physical activity was moderated by sociodemographic factors. Interaction terms were retained only if model comparisons determined significant improvement of model fit.

All three physical activity variables (domains) were zero-inflated, with approximately half of the sample reporting 0 min per week of activity in each. As such, we conducted two sets of analyses. First, we dichotomized activity in each domain (0 = none reported; 1 = any activity reported) to examine how rural–urban location and sociodemographic factors were associated with the likelihood of reporting any domain-specific activity using binary logistic regression. Second, we examined the sub-samples reporting > 0 min per week of activity in each domain to assess how rural–urban location and sociodemographic factors were associated with *how much* activity was reported using ordinary least squares regression.

The distribution of > 0 min per week of activity in all three domains was positively skewed. To account for this, we conducted a natural logarithm transform of the data for the > 0 min per week sub-samples. Regression analyses were conducted using this transformed variable, and least-squares models were examined for influential cases per Statistics Canada recommendations for weighted analysis [23]. In any case where the removal of influential cases from the model resulted in a change of > 10% in the regression estimates, results are reported for models with these cases removed. The significance and general pattern of outcomes was unchanged by the removal of influential cases in all instances.

## Results

### Participants

Study participants were mean age 47.4 years, 50.2% male, and 82.5% of the sample lived in an urban community (Tables 2 and 3). Overall unadjusted summary data of the percentage of the sample reporting any minutes per week of activity in each domain is summarized in Table 4, and the minutes per week of activity in each domain is presented in Table 5.

**Table 2:**
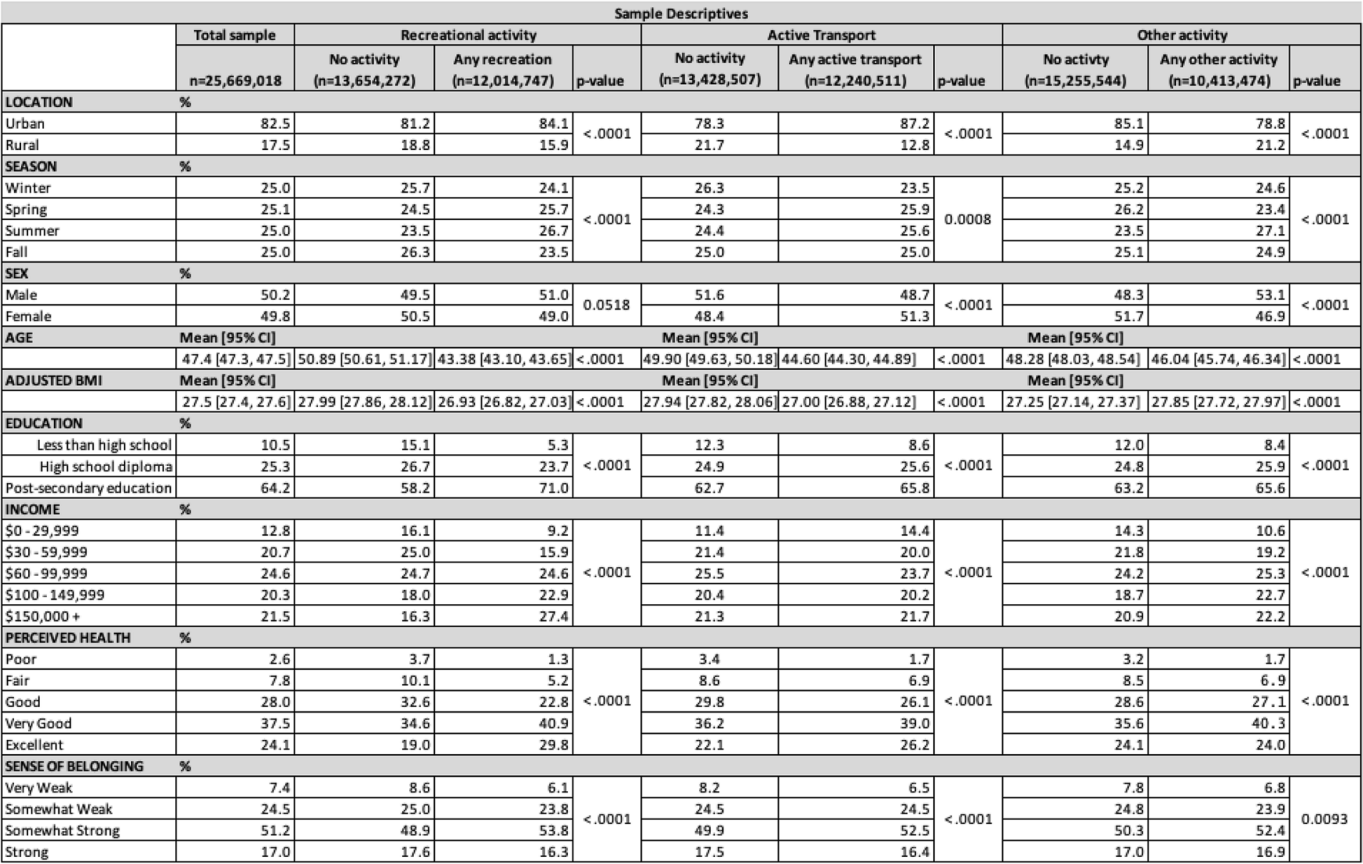
Descriptive summary of sociodemographic variables by activity level. Each above-zero (any) activity subsample is distinct. *P*-values reflect weighted chi-square or t-test outcomes between no activity and any activity sub-groups

**Table 3:**
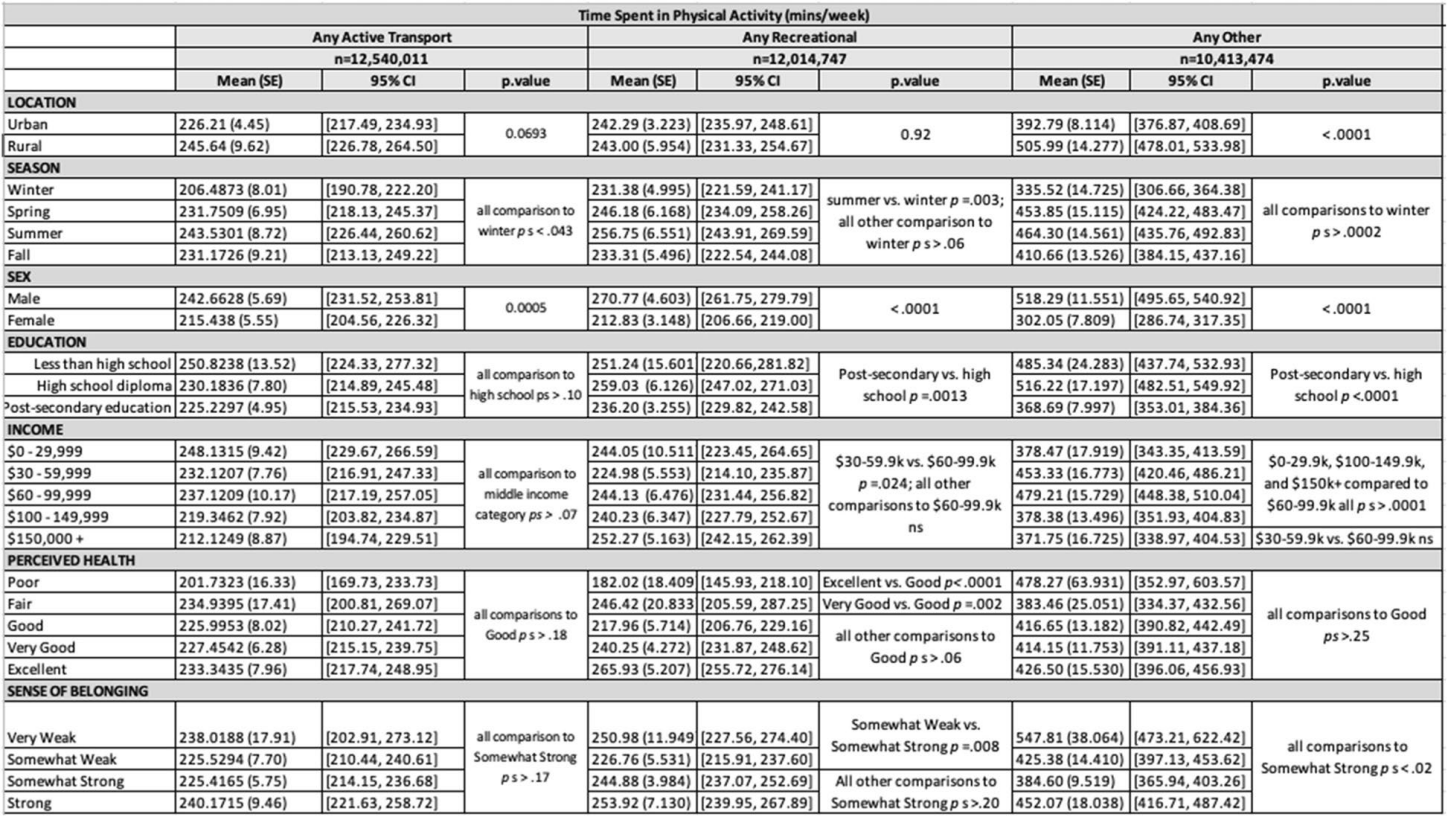
Minutes per week of activity for individuals (above-zero minutes subsamples only). *P*-values are produced by weighted chi-square test for categorical outcomes and weighted t-test or regression for continuous variables

**Table 4:** Summary of reporting no activity compared to any activity by location and sex

% OF SAMPLE REPORTING ANY ACTIVITY VS. NO ACTIVITY IN EACH DOMAIN				
	**URBAN (*n* = 21,188,895)**		**RURAL (*n* = 4,480,123)**	
	**males (*n* = 10,647,897)**	**females (*n* = 10,540,998)**	**males (*n* = 2,243,666)**	**females (*n* = 2,236,457)**
**Active Transportation**				
None	51.1	48.1	66.6	63.6
Any active transportation	48.9	51.9	33.4	36.4
**Recreational activity**				
None	50.5	54.2	61.8	53.0
Any recreational activity	49.5	45.8	38.2	47.0
**Occupational/household activity**				
None	59.6	62.9	45.3	56.2
Any occupational/household activity	40.4	37.1	54.7	43.8

**Table 5:** Summary of minutes per week of activity (above-zero minutes only) by location and sex

MINS PER WEEK OF ACTIVITY IN EACH DOMAIN (> 0 min only)				
	**URBAN**		**RURAL**	
**Active transportation**				
	**males (*n* = 5,209,725)**	**females (*n* = 5,466,640)**	**males (*n* = 749,132)**	**females (*n* = 815,014)**
Mean (SE)	233.370 (6.114)	219.384 (6.229)	307.287 (16.925)	188.970 (9.453)
95% CI	[221.387, 245.354]	[207.176, 231.592]	[274.115, 340.459]	[170.443, 207.498]
**Recreational activity**				
	**males (*n* = 5,274,923)**	**females (*n* = 4,831,692)**	**males (*n* = 856,893)**	**females (*n* = 1,051,238)**
Mean (SE)	267.845 (5.200)	214.393 (3.642)	288.794 (10.431)	205.664 (5.936)
95% CI	[257.653, 278.037]	[207.254, 221.532]	[268.350, 309.238]	[194.030, 217.299]
**Occupational/household activity**				
	**males (*n* = 4,297,269)**	**females (*n* = 3,909,152)**	**males (*n* = 1,228,039)**	**females (*n* = 979,014)**
Mean (SE)	480.541 (13.147)	296.327 (9.133)	650.366 (22.796)	324.897 (13.355)
95% CI	[454.773, 506.309]	[278.427, 314.226]	[605.686, 695.046]	[298.721, 351.073]

### Active transportation

#### Any

**Fig. 1:**
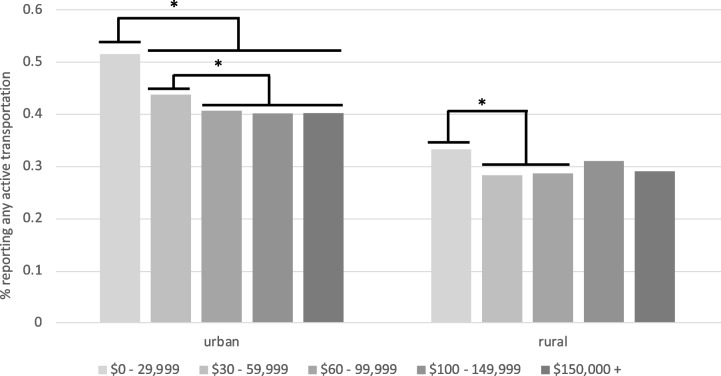
Location X Income predicting the likelihood of reporting any active transportation. Urban residents were significantly more likely to report engaging in active transportation compared to rural residents at all income levels (all *p*s < .0001)

Overall, 33.4% of rural males and 36.4% of rural females reported any minutes of active transportation per week, compared to 48.1% of urban males and 51.9% of urban females. There was a significant bivariate relationship between rural–urban location and the odds of reporting any active transportation in the best-fitting model (OR = 0.59, 95% CI [0.51, 0.67], *p* < 0.0001), along with a location X income interaction. Urban residents were significantly more likely to engage in active transportation compared to rural residents at all income levels (all *p*s < 0.0001; see Fig. 1). For urban residents, those in the lowest income category ($0–29,999/year) were significantly more likely to engage in active transportation compared to urban residents in all other income categories (all *p*s < 0.0001). Urban residents in the $30–59,999/year income category were also significantly more likely to engage in active transportation compared to all higher income categories (all *p*s < 0.02). Within the middle-income category ($60–99,999/year), there were no differences in the likelihood of engaging in active transportation compared to the remaining, higher-income categories, which also did not differ from one another (all *p*s > 0.60). In contrast, rural residents in the lowest income category were significantly more likely to engage in active transportation compared to the $30–59,999/year (*p* = 0.01) and the $60–99,999/year (*p* = 0.02) income groups but did not differ in the likelihood of engaging in active transport compared to the remaining higher-income groups (all *p*s > 0.50).

#### Minutes/week

**Fig. 2:**
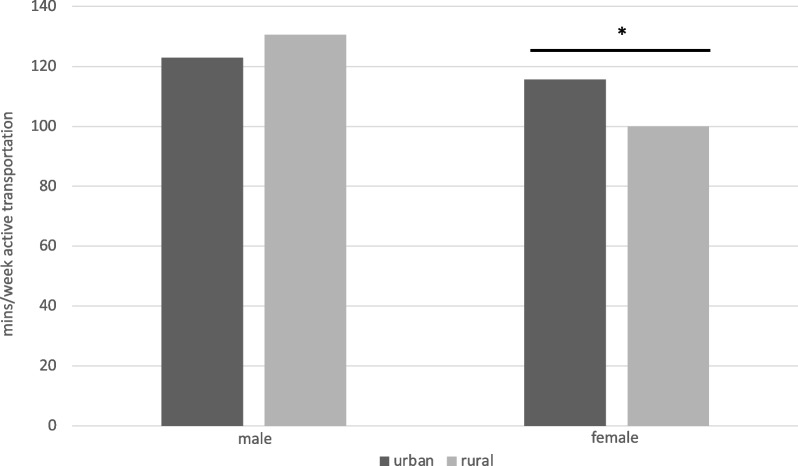
Location X Sex predicting minutes per week of active transportation (above-zero minutes only)

In the best-fitting model, there was a significant interaction between sex, rural–urban location, and active transportation. Rural–urban location was not associated with minutes per week of active transportation for males (*b* = 0.060, *SE* = 0.047, 95% CI [-0.033, 0.153], *p* = 0.206), but was associated with a small but significant difference in minutes of active transport for females (*b* = -0.145, *SE* = 0.035, 95% CI [-0.214, -0.076], *p* < 0.0001; Fig. 2). Urban females reported approximately 7 additional minutes per week of active transport compared to rural females ((*M* = 115.6 min/week vs. *M* = 99.6 min/week).

### Recreational physical activity

#### Any

**Fig. 3:**
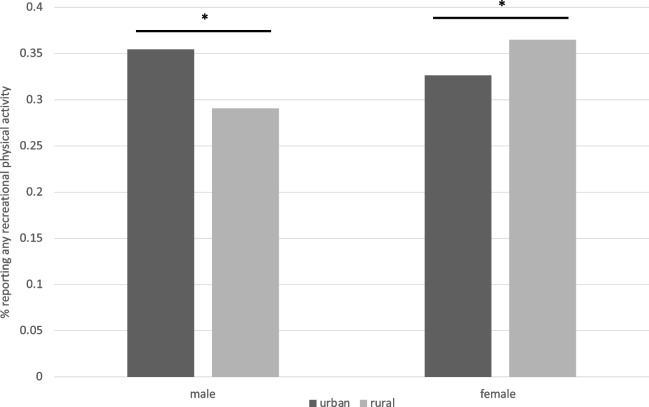
Location X sex predicting the likelihood of reporting any recreational physical activity

Overall, 38.2% of rural males and 47.0% of rural females reported any engagement in recreational physical activity, compared to 49.5% of urban males and 45.8% of urban females. There was a significant association between location and the likelihood of reporting any engagement in recreational activity in the best fitted model, along with a 2-way interaction with sex. Rural males were significantly less likely to report engaging in any amount of recreational physical activity compared to urban males (OR = 0.75, 95% CI [0.67, 0.83], *p* < 0.0001; Fig. 3). Rural females were significantly more likely to report engaging in any recreational physical activity (OR = 1.19, 95% CI [1.08, 1.30], *p* = 0.0002) compared to urban females. For urban residents, females reported a significantly lower odds of engaging in recreational activity compared to males (OR = 0.88, 95% CI [0.82, 0.95], *p* = 0.002). For rural residents, females were significantly more likely than males to report engaging in recreational activity (OR = 1.40, 95% CI [1.25, 1.57], *p* < 0.0001).

#### Minutes/week

**Fig. 4:**
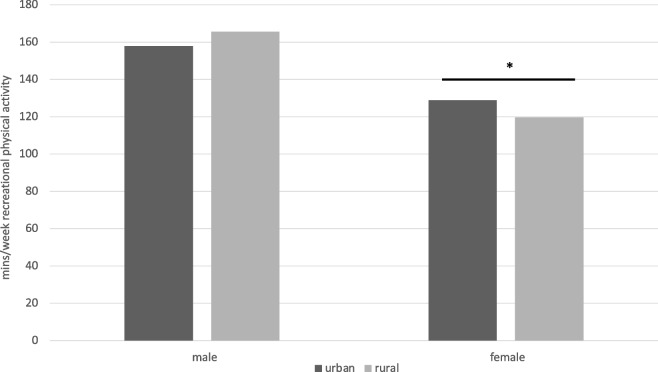
Location X Sex predicting minutes per week of recreational physical activity (above-zero minutes only)

In the best-fitting model (*n* = 7 influential cases removed), there was a 2-way interaction between rural–urban location and sex (Fig. 4). For males, there was no significant association between rural–urban location and minutes per week of recreational physical activity (*b* = 0.047, *SE* = 0.031, 95% CI [-0.014, 0.108], *p* = 0.133). For females, there was a significant effect of location (*b* = -0.075, *SE* = 0.030, 95% CI [-0.134, -0.015], *p* = 0.014), corresponding to an average of approximately 10 fewer minutes per week of recreational physical activity for rural (*M* = 119.6 min/week) compared to urban (*M* = 128.8 min/week) females.

### Occupational and household physical activity

#### Any

**Fig. 5:**
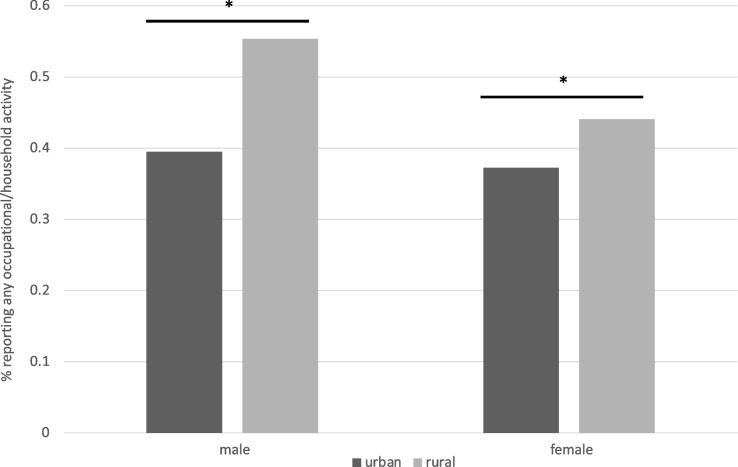
Location X Sex predicting the likelihood of reporting any occupational or household physical activity

For occupational or household activity, 54.7% or rural males and 43.8% of rural females reported any participation, compared to 40.4% of urban males and 37.1% of urban females. There was a significant association between location and the odds of reporting any occupational/household physical activity in the best-fitting model, along with a 2-way interaction with sex (Fig. 5). Rural males were significantly more likely to report engaging in any occupational/household physical activity compared to urban males (OR = 1.90, 95% CI [1.74, 2.07], *p* < 0.0001). Rural females were significantly more likely than urban females to report engaging in occupational/household physical activity (OR = 1.33, 95% CI [1.21, 1.46], *p* < 0.0001). In both urban and rural residents, females were less likely to report engaging in any amount of occupational/household compared to males (urban: OR = 0.91, 95% CI [0.85, 0.97], *p* = 0.006; rural: OR = 0.64, 95% CI [0.57, 0.71], *p* < 0.0001).

#### Minutes/week

**Fig. 6:**
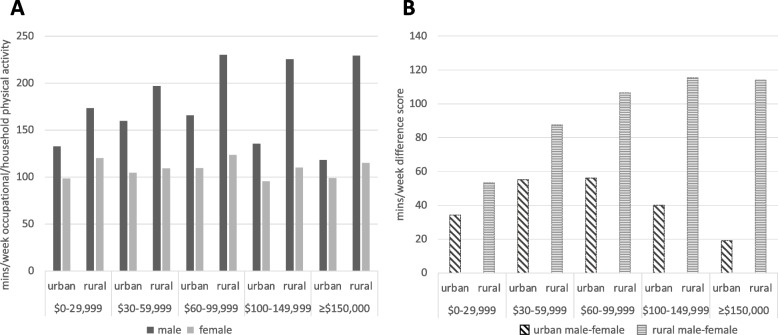
Location X Sex X Income predicting minutes per week of occupational or household activity (above-zero minutes only). Panel **A** shows minutes per week reported across the subsample; panel **B** shows the difference in minutes per week reported (male – female), highlighting a pronounced increase in sex-based differences in occupational or household activity with increasing income in rural residents

There was a significant association between location and minutes per week of occupational/household physical activity in the best-fitting model (*n* = 15 influential cases removed), qualified by a 3-way location X sex X income interaction. The effect of location was significant at all income groups (all *p*s < 0.01), and the interaction was primarily driven by the size of the rural–urban location difference across income groups (Fig. 6).

## Discussion

Our project aimed to investigate rural–urban differences in domain-specific physical activity behavior in a nationally representative sample of adults living in Canada. Additionally, we explored interactive effects of rural–urban location and sociodemographic correlates on patterns of domain-specific physical activity. Our findings demonstrate the relevance of location in predicting engagement in physical activity related to active transportation, recreational, and occupational/household physical activity. Best-fit models revealed minutes per week of physical activity behavior within each domain are explained by the interaction of location and sociodemographic variables income and sex. Our findings advance understanding of place-based discrepancies in health behaviors by specifying rural–urban differences in physical activity depend on what domain of physical activity is being evaluated and if it is measured by frequency (e.g., a binary measure of any/none) or time (e.g., minutes per week).

### Rural–urban differences in active transportation

Rural residents are less likely to engage in any active transportation compared to urban residents. Rural communities often have limited infrastructure supportive of active travel (e.g., bike lanes, sidewalks, street lighting) and increased distance to destinations compared to urban environments [24]. The physical environment is more important for active transportation than recreational physical activity and is positively associated with walking or cycling facilities [25], street connectivity, and land-use mix [26]. Our findings reflect these environmental factors and align with previous research reporting less active transportation (both minutes per week and number of trips) among a nationally representative sample of rural adolescents and adults living in the United States [27]. We observed an interaction between location and income, identifying minutes per week of active transportation decreases with increasing income. This finding aligns with previous work demonstrating with every $1000 increase in income, people are less likely to report active transportation [28]. We extended findings on the relationship between active transportation and income with finding the odds of reporting any active transportation is more strongly associated with income in urban rather than rural areas. The observed interaction between rural–urban location and income may indicate that for rural residents, active transportation is a choice (e.g., for health/fitness or environmental reasons) among those it is accessible to, whereas in urban areas, people with lower income may use active or public transit due to the high cost of vehicle ownership.

Among people in our sample reporting any active transportation, we noted sex-based differences. There were no location-based differences in active transportation for males, but rural females reported 7 min per week less than urban-dwelling females. This finding may indicate rural-residing females are obtaining their physical activity through other means, feel unsafe pursing active transportation in rural areas, or are more likely to have increased childcare responsibility (e.g., less likely to work outside the home and thus less likely to need to travel) than urban-dwelling females. The difference in minutes per week of active transportation for females was small but consistent. As an important way to meet physical activity guidelines and contribute environmental benefits, strategies to encourage active transportation for rural residents may consider policy factors (e.g., beyond the individual) by focusing on supporting appropriate infrastructure for rural communities and increasing perceptions of safety (e.g., appropriate streetlighting, reducing risk from wildlife encounters).

### Rural–urban differences in recreational physical activity

Recreational activity, often called leisure-time physical activity, includes organized sports or non-organized activity people do in their free time. Our findings indicate a rural–urban difference in odds of participating in any recreational activity. Among people who do any recreational activity, rural females participate in 10 min per week﻿ less than urban-dwelling females. Less participation in recreational physical activity reported by rural residents aligns with our previous work indicating Canadian rural adults are four times as likely than urban residents to report limited access to recreation facilities [5] and work in the United States indicating rural residents achieve less of their physical activity in the leisure-time domain [16].

Interesting sex differences emerged in our analyses of recreational activity. While males in rural areas were less likely to report any engagement in recreational activity, those who did participate in recreational activity didn’t differ from urban males in minutes per week spent being active. In contrast, rural females were more likely than urban females to report any amount of recreational activity, but among those who participated in recreational activity, rural females reported fewer minutes per week. These findings may suggest frequency of engagement in an activity is dissociable from time spent in activity differently for males and females. Reasons for this finding are only speculative, but females may be more likely to find time for recreational activity, but perhaps less time due to other responsibilities.

### Occupational and household physical activity

Rural active living environments are characterized by interest in productive work-related and home-based pursuits, outside of what is traditionally considered and promoted as exercise or physical activity (e.g., performance-based fitness; [29, 30]). Our findings indicate rural males and females are more likely to report any occupational or household physical activity than urban males and females, extending previous work on physical activity patterns of rural and urban adults in the United States [15, 16]. The sex X location interaction was stronger for males, suggesting how much occupational or household physical activity (minutes per week) people engage in is heavily dependent on sex and income. For rural residents it may be likely people earning higher income are working in physically intensive industrial jobs, whereas in urban areas higher-income households are less likely to be working physically demanding jobs, and thus engage in more recreational physical activity. This theory is further supported by previous findings of people in physically active occupational groups tending to perform less leisure-time physical activity [31] with work demands and physical demanding work cited as reasons for not engaging in recreational activity [32].

The relationship between occupational physical activity and mortality in males may indicate an important health risk for rural residents [33, 34]. While some previous work has suggested the relationship between higher occupational activity and poor health outcomes is tied to income (e.g., lower income males have fewer options for less demanding work; [35]), our work suggests increased occupational physical activity is consistent across rural males of all income categories. Although we do note the survey question did not differentiate between occupational and household activity, given the discrepancy in life expectancy between rural and urban males in Canada [1], exploring relationships between occupational demands, physical activity, rurality, and mortality is an important area for further study.

### Sex-based differences in patterns of physical activity

In addition to observed location-based differences in patterns of domain-specific physical activity, we noted several sex-based differences and interactions. Regardless of location or income, males engaged in more active transportation, recreational physical activity, and occupational/household physical activity than females. Worldwide, women tend to report a lower amount of moderate-to-vigorous physical activity compared to men [36, 37]. Previous work exploring domain-specific differences in physical activity across low- middle-and high-income countries identified women tend to achieve less physical activity through occupational/household activity and more through travel [12]. Conversely, in a sample of rural residents in the United States, female participants engaged in less physical activity across all domains [17]. The current findings extend our previous work demonstrating the interactions between rural–urban location and self-identified sex in meeting physical activity guidelines [4] and suggest both the amount and types of physical activity differ based on location and sex and should be considered in health promotion strategies.

While our reporting on sex-based differences in physical activity and domain-specific patterns of activity adds insights, we note this is based on a binary self-reported measure of sex (options male or female) and any associations with gender related constructs (e.g., gender roles/norms) are assumptions. This is a limitation of the CCHS data set, which has been changed in more recent data collection cycles to include questions regarding sex at birth and gender identity [38]. Future work should consider both sex and gender variables in understanding physical activity behavior between rural and urban communities, explore physical activity experiences of people outside of a cisgender binary, and how sex and gender variables interact with other social categories to impact behavior.

### Limitations

We did not conduct a full-sample analysis for each domain of physical activity accommodating the zero-inflated data (e.g., a Poisson regression). Instead, we opted to split the sample into a binary of 0 min and > 0min/week to conduct separate analyses. There are distinct sub-samples for each domain of physical activity as only approximately 20% of the sample reported no activity in all three domains and within each domain the sample is not necessarily directly comparable to the sample in a different domain. However, conducting an analysis on the odds of reporting any physical activity in each domain in addition to comparing how many minutes provides some data that can be used to explore patterns of behavior and what predicts reporting no activity in each domain.

We observed some exclusion bias among females, those with less than secondary school education, lower income, poorer perceived health, and those with a weaker sense of belonging to their community (see Supplementary file). These exclusions were based on incomplete data sets, likely related to bias in survey design or conduct, and indicate our sample may not be representative of the wider Canadian population.

## Conclusions

People living in rural communities are less likely to engage in active transportation and are more likely to participate in occupational or household activities compared to urban residents. There are sex-based differences in physical activity patterns across rural–urban location with rural females reporting more recreational physical activity and rural males higher contribution from occupational/household physical activities. Overall, findings suggest different physical activity patterns for rural and urban dwelling adults living in Canada. Considering the multiple interactive effects of place and sociodemographic categories are necessary for an equitable approach to physical activity promotion and in analyses of behavioral patterns.

### Supplementary Information

**Additional file 1.**

## Data Availability

The data supporting the findings are from the Canadian Community Health Survey (2017 annual cycle). Data are available from Statistics Canada and accessible through the Canadian Research Data Centre Network (https://crdcn.ca/). No direct link to the dataset is available outside of the Research Data Centre.
