# Supplementary material for: Work, travel, or leisure: comparing domain-specific physical activity patterns based on rural–urban location in Canada
Source: BMC Public Health. 2023 Nov 10;23:2216. doi: 10.1186/s12889-023-16876-1 (PMC10637018; doi:10.1186/s12889-023-16876-1)
Supplement: Supplementary file 1 — Additional file 1. [file 12889_2023_16876_MOESM1_ESM.pdf]

Supplementary file – Comparison of included and excluded participants.

		Included (unweighted N=47,266)	Excluded (unweighted N=5274)	Comparison
		Mean (SE) / %	Mean (SE) / %	
Age	Mean	47.4 (0.06)	53.6 (0.41)	$p < .001$
BMI	Mean	27.5 (0.04)	Data missing	N/A
Location	Urban	82.5	83.5	$p = .177$
	Rural	17.5	16.5	
Sex	Male	50.2	40.6	$p < .001$
	Female	49.8	59.4	
Season	January-March	25.0	47.1	$p = .693$
	April-June	25.1	47.6	
	July-September	25.0	49.2	
	October-December	25.0	50.8	
Education	Less than secondary school	10.5	21.4	$p < .001$
	Secondary school	25.3	24.8	
	Post-secondary+	64.2	53.8	
Income	$0-29,999	12.8	15.5	$p < .001$
	$30-59,999	20.7	26.6	
	$60-99,999	24.6	24.2	
	$100-149,999	20.3	18.2	
	$150,000+	21.5	15.5	
Perceived Health	Poor	2.6	9.1	$p < .001$
	Fair	7.8	13.9	
	Good	28.0	31.4	
	Very Good	37.5	27.1	
	Excellent	24.1	18.4	
Sense of Belonging to Community	Very Weak	7.4	9.6	$p = .018$
	Somewhat Weak	24.5	22.1	
	Somewhat Strong	51.2	50.1	
	Very Strong	17.0	18.2	
